# S-100 B Concentrations Are a Predictor of Decreased Survival in Patients With Major Trauma Independently of Head Injury

**DOI:** 10.1186/2197-425X-3-S1-A31

**Published:** 2015-10-01

**Authors:** CA Pfortmueller, C Drexel, S Krähenmann-Müller, AB Leichtle, GM Fiedler, G Lindner, AK Exadaktylos

**Affiliations:** Vienna General Hospital and University of Vienna, Clinic for General Anesthesiology, Intensive Care and Pain Managment, Vienna, Austria; University Hospital and University of Bern, Department of Emergency Medicine, Bern, Switzerland; University Institute of Clinical Chemistry, Inselspital-Bern University Hospital, Centre of Laboratory Medicine, Bern, Switzerland; Hirslanden - Klinik im Park, Department of Emergency Medicine, Zurich, Switzerland

## Principals

Major trauma remains one of the principle causes of disability and death throughout the world. There is currently no satisfactory risk assessment to predict mortality in patients with major trauma. The aim of our study is to examine whether S-100 B protein concentrations correlate with injury severity and survival in patients with major trauma, with special emphasis on patients without head injury.

## Methods

Our cross-sectional data analysis comprised adult patients admitted to our emergency department with a suspected major trauma between 1.12. 2008 and 31.12 2010. S-100 B concentrations were assessed routinely in major trauma patients.

## Results

A total of 378 (27.7%) of all patients had major trauma. The median ISS was 24.6 (SD 8.4); 16.6% (63/378) of the patients died.S-100 B concentrations correlated overall with the ISS (p < 0.0001). Patients who died had significantly higher S-100 B concentrations than survivors (8.2 µg/l versus 2.2 µg/l, p < 0.0001). Polytraumatised patients with and without head trauma did not differ significantly with respect to S-100 B concentration (3.2 µg/l (SD 5.3) versus 2.9 µg/l (SD 3.8), respectively, p = 0.63) or with respect to ISS (24.8 (SD 8.6) versus 24.2 (SD 8.1), respectively, p = 0.56). S-100 B concentrations correlated with survival (p < 0.0001) in all patients and in both subgroups (p = 0.001 and p = 0.006, respectively).

## Conclusions

S-100 concentrations on admission are of considerable diagnostic value in the evaluation of injury severity and survival of major trauma patients.

S-100 B concentrations are not significantly different in major trauma patients with and without head injury. Death is associated with increased S-100 B concentrations, regardless of concomitant head trauma.

